# Digital Gaming for Improving the Functioning of People With Traumatic Brain Injury: Protocol of a Feasibility Study

**DOI:** 10.2196/resprot.4841

**Published:** 2016-02-09

**Authors:** Maritta Välimäki, Jyrki Korkeila, Kaisa Kauppi, Johanna K Kaakinen, Suvi Holm, Jukka Vahlo, Olli Tenovuo, Heikki Hämäläinen, Jaana Sarajuuri, Pekka Rantanen, Tage Orenius, Aki Koponen

**Affiliations:** ^1^ Faculty of Medicine Department of Nursing Science University of Turku Finland; ^2^ Turku University Hospital Turku Finland; ^3^ Faculty of Medicine University of Turku Turku Finland; ^4^ Hospital District of Satakunta Harjavalta Finland; ^5^ Faculty of Social Sciences Department of Psychology University of Turku Turku Finland; ^6^ Turku School of Economics Centre for Collaborative Research University of Turku Turku Finland; ^7^ Turku University Hospital Department of Rehabilitation and Brain Trauma, Division of Clinical Neurosciences Turku Finland; ^8^ University of Turku Department of Neurology Turku Finland; ^9^ Validia Rehabilitation Helsinki Helsinki Finland; ^10^ ORTON Ltd ORTON Foundation Helsinki Finland

**Keywords:** digital games, brain injury, cognitive rehabilitation

## Abstract

**Background:**

Traumatic brain injury (TBI) is a critical public health problem. The recovery process for people with TBI is typically slow and dependent on complex and intensive assisted rehabilitation programs.

**Objective:**

To evaluate the effects and feasibility of digital games for cognitive functioning and general well-being among people with traumatic brain injury.

**Methods:**

This is a single-site feasibility study conducted in Finland, which uses a pragmatic, randomized controlled trial with three arms, and will recruit patients from the Turku University Hospital, Division of Clinical Neurosciences in Finland. Participants must meet the following inclusion criteria: (1) a Finnish speaking adult, aged 18-65 years; (2) diagnosed with a traumatic brain injury (diagnostic criteria ICD-10, S06.X, T90.5) in the University Hospital; (3) access to a TV, a computer, and the Internet at home; (4) not an active digital gamer (5 hours or less a week); (5) willing to participate in the study. Participants must have been discharged from the neurologic treatment period for traumatic brain injury for over 12 months before the commencement of the trial, and they may not have actively participated in cognitive rehabilitation during the 3 months prior to the trial. Written informed consent will be mandatory for acceptance into the trial. Exclusion criteria are as follows: (1) sensory, cognitive, or physical impairment (eg, severe cognitive impairment); (2) a deficiency restricting the use of computers or computer game control system unaided (eg, impairment in vision, severe astigmatism, hemiplegia, disorder in visuospatial perception, dysfunction of the central vestibular system); (3) apathy identified in previous neuropsychological evaluations; (4) diagnosed severe mental disorders (eg, schizophrenia or severe depressive disorders to be identified in medical records as the secondary diagnosis).

**Results:**

The preparatory phase for the study is fulfilled. Recruitment started in June 2015 and finished November 2015. Results will be reported in 2016.

**Conclusions:**

The specific outcomes such as primary outcome measures were selected because they are widely used psychological tests and thought to be sensitive to changes in the cognitive functions related to TBI.

**Trial Registration:**

Clinicaltrials.gov NCT02425527; https://clinicaltrials.gov/ct2/show/NCT02425527 (Archived by WebCite at http://www.webcitation.org/6esKI1uDH)

## Introduction

Traumatic brain injury (TBI) is a critical public health problem. In the USA alone at least 5.3 million citizens live with disabilities resulting from TBI [1]. In Finland there are about 40,000 people with TBI [2] and approximately 100,000 people live with disabilities resulting from brain injuries [3]. The most common groups with brain injuries are teenagers and young adults between the ages of 15 and 34. Treatment of TBI is long lasting with long-term care, which therefore incurs economic cost to health systems [4]. Impairments, apathy, mood disorders, impulsivity, and changes in personal character [3] or deficits of attention and short-term memory are among the most common and disabling characteristics of people with TBI [5]. The recovery process is typically slow and depends on complex and intensive assisted rehabilitation programs [6-8].

Although physicians have long believed that individuals with brain injury benefit from early and long-standing therapeutic intervention [9], direct evidence is still scarce [10]. A recent expert panel on cognitive rehabilitation suggested that metacognitive strategy training focusing on functional everyday activities is appropriate for people with TBI [11]. A systematic review by Spreij et al suggests that computer-based cognitive retraining is considered to be the most promising novel approach of the last decade, because of the positive results in improving memory function after an acquired brain injury [12]. Training with video games is also a rapidly developing area for industry, since it has been estimated that the average young person has played a total of 10,000 hours of games by the age of 21 [13]. Virtual reality games have been found to improve patients’ mobility [14] or motor learning [15]. A meta-analytic study by Toril et al [16] indicated based on 21 experimental studies and 474 trained and 439 healthy older controls that video game training produces positive effects on cognitive functions, including reaction time, attention, memory, and global cognition. However, inferences from the results have to be made cautiously because of the problem of the high heterogeneity of the studies. Further, a systematic review concluded that video games have the potential to improve health outcomes in psychological and physical therapy [17]. On the other hand, Lampit and colleagues [18] found that computerized cognitive training in healthy adults is modestly effective at improving cognitive performance, but efficacy varies across cognitive domains and is largely determined by design choices.

Some encouraging results for virtual reality interventions have been observed in patients with post-stroke conditions [19]. For example, those who are active players show better performance in alertness and cognition compared to those who do not play games [20,21]. In addition, the game-based exercises in recovery process have shown increased motivation to perform rehabilitative tasks [22]. Further, preliminary results have shown that playing action video games has produced significant improvements in attentional control for healthy adults [23].

Still the scientific evidence for this area is at best mixed. Ball and colleagues [24] conducted a large-scale cognitive training study and found that when memory, attention, and problem solving were trained independently, trainees improved each skill respectively. However, there was no transfer to other untrained skills. Game programs were also found to be inadequate for efficient integration in current clinical practice [25,26]. Stand-alone systems without any human interaction have also been criticized [27]. In addition, there is still a lack of information regarding what kinds of games participants want to play as well as the effectiveness of serious games for patients with brain injury. Gravel and colleagues [28] concluded in their systematic review that there is a paucity of well-designed clinical studies for patients who sustain mild TBI. The large variability in outcomes measured may also limit comparison between studies. Although experiments using games among people with brain injury have been conducted, the effectiveness of commercially available games has been evaluated to a lesser extent [19]. As traumatic brain injury causes long-term disability with adverse social, psychological and economic consequences, it is therefore important to seek methods to optimize independence and social participation to reduce long-term care needs and enhance quality of life among people with traumatic brain injury [29]. The meta-analyses by Lohse and colleagues [19] also indicate that more evidence is needed to evaluate the effectiveness of games among people with brain injury.

The aim of this study is to evaluate the effects and feasibility of digital games for cognitive functioning and general well-being among people with traumatic brain injury.

## Methods

### Trial design

This is a single-site feasibility study, conducted in Finland, which uses a pragmatic, user-centered, randomized controlled, three-arm parallel-group design.

### Participants

Patients will be individually and randomly assigned to one of the three parallel groups, initially in a 1:1:1 ratio, to an intervention group (rehabilitation gaming), active control group (entertainment gaming) or passive control group (no gaming) organized by the research study.

To be eligible for this study, participants must be Finnish speaking and reading adults between the ages of 18 and 65, who meet the eligibility criteria for people with traumatic brain injury (diagnostic criteria ICD-10, S06.X, T90.5) Participants must have been discharged from the neurologic treatment period due to traumatic brain injury over 12 months before the recruitment and must not have been actively participating in cognitive rehabilitation during the last 3 months. They must own a TV, a computer and have internet access at home, but must not be active digital gamers (5 hours or less a week). Potential participants will not be admitted to the study if they have any of the following exclusion criteria: sensory (eg, serious visual impairment), cognitive (eg, memory problems, slow processing speed, a lack of attention, linguistic problems) or physical impairment (eg, severe cognitive impairment or impairment in vision, severe astigmatism, hemiplegia, disorder in visuospatial perception, dysfunction of the central vestibular system), which may restrict the use of computers or computer game control system unaided; apathy identified in previous neuropsychological evaluations; a diagnosis of a severe mental disorder (eg, schizophrenia or severe depressive disorders to be identified as the secondary diagnosis); or having been active in cognitive rehabilitation during the previous 3 months. The primary source to assess patient eligibility will be the university hospital electronic medical records and later telephone calls and interviews with a trained psychologist.

The study will take place at the Turku University Hospital, Division of Clinical Neurosciences (Operational Division of Clinical Neurosciences, Department of Rehabilitation and Brain Trauma, in collaboration with former neurologic outpatient clinic and rehabilitation units of the university hospital) located in southern Finland. The Division is specialized in neurological and psychiatric inpatient rehabilitation, inter-professional neurological education, rehabilitative examinations, and the inpatient and outpatient care of traumatic brain injuries. Each year, about 350 patients are admitted to the Division services units [30].

The randomization (started in June, 2015) will target patients who have been registered in the Turku University Hospital electronic medical records. The recruitment will focus on patients who have been discharged from the Turku University Hospital, Division of Clinical Neurosciences at least 12 months before the recruitment. It has been indicated that using games in longer interventions will be facilitated more easily if training is provided in the patient’s own environment, such as home, due to easier access and reduced impact on school or work activities [31]. To capture a representative sample of patients, the Turku University Hospital electronic medical records will be accessed by the authority of the chief medical doctor of the Hospital together with the Research Assistant. All patients with a TBI diagnosis will be screened to determine which patients fulfill the eligibility criteria for study participation.

### Interventions

Patients will be randomly assigned to rehabilitation gaming (intervention group), entertainment gaming (active control group), or passive control group (no gaming).

First, the rehabilitation gaming (intervention group) will use an internet browser-based digital brain training program, CogniFit [32]. This web-based cognitive training platform includes about 33 games designed with the purpose of improving the user's cognitive abilities as brain exercises. In this study, we aimed at controlling for the type of “cognitive demands” the games pose to the player by preselecting games that share certain features while allowing participants to choose a game that they find pleasurable to engage with. For the purpose of the research, a selection of games will be included in the intervention. The game tasks target different cognitive abilities such as memory, visual-spatial ability, attention, tracking and fast decision making. The intervention in our study is user-centered. This means that participants are instructed to play at least one exercise from each of the four categories during each training session daily; they are otherwise free to choose which exercises they wish to play. It is reasonable to assume that in real life people would choose to play games that they like. The possibility to choose a game that you like is supposed to increase the likelihood that participants engage in gaming as instructed. The game tasks can also be adapted to players’ skills getting more difficult as the players progress. CogniFit requires a computer with Internet access.

The participants will have their own user account on CogniFit, which will record the games they have selected, how often and how long their playing session has taken, and how they have progressed in each game. Access into CogniFit will be introduced to the participants during the introductory meeting by the Research Assistant. To assess the amount of time Research Assistants are spending with each participant, the length of the introductory meeting will be recorded. This will ensure that we are aware of the level and time of support offered to each participant before the intervention start. Any help later at home or by telephone will be offered by a Technical Assistant if needed; any contacts with the Technical Assistant are recorded. The participants will be guided to use the rehabilitation gaming for at least 30 minutes per day over a period of 8 weeks. Daily gaming sessions are supported by studies [13,33,34], although opposite suggestions have also been found in the literature. For example, the meta-analytic study by Lampit and colleagues [18] concluded that training more than 3 times per week in an unsupervised home environment was specifically ineffective for healthy older adults. To encourage, motivate, and hold participants to training in each category, they will be supported in planning a schedule for their training sessions (days, time, and frequency) by the Research Assistants for the entire 8 week gaming period. The participants will also be encouraged to seek help and support by telephone. If requested by the participant, a technical assistant will visit the participant’s home to help set up the computer in a quiet place and offer training [35].

Further, to support the participants’ gaming activities, their ability and previous experience in playing digital games will be explored. This will ensure that the participants have basic gaming skills required for active gaming. A description of the overview of the program will be offered. A new email address, the passwords for the email account and personal account will be generated. This is necessary because the browser-based program requires access through a website and the user will log in with an email address and a specified password. The participant will also experiment with the game unaided to find out possible barriers in his/her gaming. The program will record participants' progression and scores on each of the games. In order to do this, the research team will have access to the program to monitor the progress of each participant’s game scores. Information about the frequency of training sessions will also be recorded by the participants themselves into a specific calendar. Based on the information in the diary, we can statistically control for the effect of actual amount of gaming on the results. Beside the participants’ diary, participants’ adherence to and motivation [36] for gaming will be supported and monitored by weekly telephone calls by the research assistants.

Second, the active control group, (ie, “entertainment gaming”) will use commercial digital games designed for Sony Playstation 3 (PS3) consoles. PS3 consoles will be connected to the participant’s TV systems via a HDMI cable or an RCA connector cable; using a console does not require an internet connection. The games will be played with wireless and rechargeable Sony DualShock gamepad controllers. As in the intervention group, the participants chose a game that they found pleasurable to engage with. The possibility to choose a game may increase the likelihood that participants engage in gaming as instructed. Participants will have already selected the most favored game at home out of eight possible games. Entertaining games were carefully selected to correspond to the CogniFit games. Although the eight games differ from each other with respect to aesthetics and narrative qualities, each game contain the same core gameplay. Gameplay can be defined as consisting of two elements: the challenges the designed game presents for the player as the game proceeds and the actions the player decides to take to overcome the challenges and to reach in-game goals [37]. The core gameplay of each of the eight games consist of: 1) real-time action which demands quick responses and decisions; 2) identifying relevant information from irrelevant noise; 3) targeting, tracking and shooting; 4) orienting and navigation in 3D space; and 5) tasks that require memorizing and reasoning.

The attractiveness of the game for the player will also be ensured. This, in turn, is thought to increase the motivation to engage in training. We thought that it is important that participants are not forced to play, for example, violent games if they prefer not to. It is reasonable to assume that in real life, people would choose to play games that they like.

The game selected will be played together with the Research Assistant during the introductory meeting. To assess the amount of time Research Assistants are spending with each participant, and the needs depending on the type of console, the length of the introductory meeting will be recorded. This will ensure that we are aware of the level and time of support offered to each participant. As with the intervention group, ability to play digital games will be explored to ensure that participants have the basic gaming skills required for active gaming. An overview of the use of the PS3 console will also be offered and a tutorial demonstration will be given. This will include, for example, how to start the console, how to play the game, how to use the controller, how to change game options (ie, game difficulty and speed). The participant will be able to try the game that they have preselected from the list of eight games. In addition, the project will purchase the game for the participant either by buying the game from 1) the official Playstation Store and downloading and installing the game into a new PS3 console that will be offered to the participant for intervention use at home or 2) a retail store. If requested by the participant, a Technical Assistant will visit the participant’s home to help set up the console in a quiet place and offer training [35] or guidance by telephone. As with the intervention group, the participants will be guided to play the console for at least 30 minutes per day over a period of 8 weeks [13]. To assess the amount of time Research Assistants are spending with each participant, the length of the introductory meeting will be recorded. This will ensure that we are aware the level and time of support is offered to each participant before the intervention start. Any help later at home or by telephone will be offered by the Technical Assistant if needed; any contacts with the Technical Assistant are recorded. To encourage, motivate, and hold participants to a training schedule, participants will be supported in planning their training session schedule (days and times) with the Research Assistant for the entire 8 week gaming period. Information about game sessions (day, time, frequency, play progress) will also be recorded by the participant into a calendar (gaming diary). Based on the information in the diary, we can statistically control for the effect of actual amount of gaming on the results. Further, adherence to gaming will be supported and monitored by weekly telephone calls by the Research Assistant. The participants will also be able to change the game during the 8-week period if they have concerns due to violent content, for example.

Third, the participants in the passive control group will not have gaming activities organized by the project. As with the intervention groups, the Research Assistant will call weekly to the participants in this study arm. In addition, as patients in a control group, these participants will be offered an opportunity to have games and consoles for a 2 week period free of charge after the study. Further, 5 out of the 90 participants will have a chance to randomly win a console to keep after the study intervention period.

The procedure for patient recruitment and allocation will consist of specific steps. First, potentially eligible participants will be selected from the hospital patient records based on the inclusion criteria with the help of a specialist in neurology. Those patients who have been screened and assessed a prior to meet the inclusion criteria will be contacted by telephone by the Research Assistant. Any cognitive and physical problems limiting patients’ ability to participate in the study will be discussed by phone. If a patient cannot be reached by the telephone call, a written information letter about the study will be posted to him or her. If interested, he or she can contact to the Research Assistant. A preliminary description of the study will be offered, and the inclusion criteria for the study will be described to the potential participants. Based on the discussion with the participants, their eligibility for the study will be decided. Eligible participants will also be asked about preliminary interest in the study. They will be informed that they will receive information about the study by post, 2 informed consent forms, questionnaires to be filled in (baseline data), and a short description of the 8 optional games (in case of allocation to the control group).

Possible participants will also be made aware that after 1-2 weeks they will receive another call from the Research Assistant. This will ensure that potential participants have enough time to read all materials, ask questions, make the decision to participate in the study or not and decide what type of game they would like to play if allocated to the group of entertainment gaming. During this telephone call, those who are willing to participate in the study will get information about the practical arrangements of the study and the place of the data collection. They will also be informed that all travel costs will be covered and that there will be a face-to-face meeting scheduled that may take 1-1.5 hours in total. In addition, the participants will be made aware that they are free to withdraw from the study at any time.

During the face-to-face meeting at the research laboratory, participants will be confirmed as successfully or unsuccessfully meeting the inclusion criteria. The current cognitive status of the participant will be initially assessed during the face-to-face meeting with the Research Assistant. After the initial interview, a battery of neuropsychological tests will be used in the baseline measurement phase (see primary and secondary outcome measures). If during testing it is obvious that the participant suffers from severe cognitive impairments that prevent the use of regular computers or games, the participant will be excluded from the study.

Eligible participants will sign an informed consent form of their own free will. Should a person consent, their previously-collected baseline data will be gathered. Cognitive measurements will be then conducted by a trained psychologist. After that, the trial manager will receive a message by email, text message or telephone about each eligible patient and will allocate the patient to one of the three arms of the trial, based on a computer generated randomization list (intervention group vs. active control group vs. passive control group). The Research Assistant will receive this information. Recruitment will continue until all required data has been received.

## Results

The preparatory phase for the study is fulfilled. Recruitment started in June, 2015 and finished September, 2015. Results will be reported in 2016.

## Discussion

### Outcomes

The specific outcomes, such as primary outcome measures (The Trail Making Test, parts of the Wechsler Adult Intelligence Scale, 4th Edition, or WAIS-IV) were selected because they are widely used psychological tests [39,42]. They were thought to be sensitive to changes in the cognitive functions typically related to TBI. Moreover, these functions were assumed to be trained by the video games. The Trail Making Test is one of the most used neuropsychological tests globally, allowing comparisons to previous studies on TBI and rehabilitation. WAIS-IV tasks also are among the most used psychological tests in the world, and they are sensitive enough to discriminate even healthy adults. WAIS-IV has been standardized also in Finland - the home country of the participants - allowing us to compare our sample to national standards. The secondary outcome measures were selected so that a comprehensive picture of the potential changes in cognitive functions that can be assumed to be trained by the games (both the video games and the cognitive training game) is obtained. Moreover, we thought it is important to record changes in mood and self-efficacy as these influence daily life of a patient. Finally, for practical purposes it is important to gain knowledge of the feasibility of the games, as measured by adherence to the treatment and the participants’ personal experience with the game.

#### Primary Outcome

##### Processing Speed and Visuomotor Tasks

- *The Trail Making Test (TMT)* requires visual search, scanning, speed of processing, mental flexibility, and executive functions [39]. The test consists of two parts. In the first part, patients are given a paper displaying circles numbered 1 to 5 in random order. The patient is told to draw lines that will connect the numbers in ascending order. In the second part, there are circles with numbers from 1 to 13 and letters from A to L. This time, the patient draws lines to connect the circles so that they alter between numbers and letters in an ascending order (for example: 1-A-2-B-3-C) and so on. The time it takes to complete the trail is measured in both parts of the test. Errors do not affect the final score, but they must be corrected by the patient [40,41].
- *WAIS-IV tasks* (symbol search, coding and cancellation*)* are a test package used to measure cognitive skills of adult patients, especially skills of sorting out simple visual information, monitoring, making progress in the task, maintaining attention, visuomotor co-ordination and visual memory [42]. The tasks constitute 4 indices that measure different areas of cognitive skills. In symbol search and cancellation tasks, the participants perform a visual search in order to find out if a certain symbol is in the midst of other symbols. In the symbol search task, the symbols are organized in rows, and the participant has to indicate whether or not the required symbol was on each row. In the cancellation task, the participant looks for the same symbols during the whole task. In the coding task, the participant is given a set of numbers and symbols that match each other. Their task is to fill out an empty grid containing only numbers with the appropriate symbols matching those numbers. The test-retest reliabilities of the tasks used in this study ranged from 0.78 to 0.86 over an 8-82 day period in the original WAIS-IV standardization study [42]. A version of the test [42] containing norms for the Finnish population is used in this study.

#### Secondary Outcomes

##### Attention and Executive Functions

A *Simon task* [43,44] will be used to measure the inhibition component of executive functions [45]. This is a computerized visuomotor task in which correct responses and reaction times are measured. In the task, a blue or red square appears on either the left or right side of the screen. The participant is instructed to push the left button on a response pad each time a blue square appears and the right button each time a red square appears, irrespective of which side the square is presented. In congruent trials, the response button is on the same side as the square, and in incongruent trials the square is on the opposite side of the response button (ie, the irrelevant spatial information is conflicting with the correct response). The differences in reaction times and error rates between the incongruent and congruent trials (the Simon effect) are used as the dependent measurements in this task. These variables reflect the extra processing cost of having to inhibit the incompatible spatial location of the stimulus.

##### Working Memory

- *WAIS-IV* (digit span) measures working memory [42]. In the first part of the task, the participants repeat numbers in the order they hear them. This task measures memory, coding, attention and auditory processing. In the second part, they repeat the numbers backwards. This task requires the use of working memory, processing information before giving an answer, internal processing and visuospatial organization. In the third part of the task, the participants repeat numbers in numerical order. This task again measures working memory and internal organization of the stimulus, but is more demanding than the other two tasks [42].
- *The Paced Auditory Serial Addition Test* (PASAT) was originally developed by Gronwall [46] to measure auditory information processing speed, flexibility and calculation skills, in order to monitor the rehabilitation of patients with mild head injuries. This study includes a well-established version of the test in which the stimulus presentation rates were adapted for MS-patients by Rao and colleagues [47]. In the first part, single numbers are presented every 3 seconds. The patient adds each new number to the last number prior to it. In the second part, the numbers are presented every 2 seconds, but the task is the same. The test score is the number of correct sums given in each trial.

##### Depression

*The Patient Health Questionnaire* (PHQ-9) [48,49] includes 9 items where respondents are asked to indicate with four-point scale how often they have been bothered by any of the problems over the last two weeks (0 = not at all; 1 = several days; 2 = more than half of the days; 3 = nearly every day). Based on the individual items, a total score is formed: the higher the score, the more severe the depression symptoms (range 0-27).

##### Self-Efficacy

*The General Self-efficacy Scale* (GSC) [49] has been created to assess a general sense of perceived self-efficacy to predict coping with daily hassles as well as adaptation after experiencing a variety of stressful life events. The scale for 10 items is self-administered and responses are made on a 4-point scale (1 = Not at all true; 2 = Hardly true; 3 = Moderately true; 4 = Exactly true). It takes about 4 minutes to complete. The final composite score ranges from 10 to 40, and comprises the sum of all 10 responses: low scores represent a lower ability to cope with daily problems.

##### Executive Functions

The Behavior Rating Inventory of Executive Function-Adult Version (BRIEF-A) is a 75-item questionnaire that focuses on executive functions in daily life [50]. The questionnaire consists of a behavioral regulation index (inhibition, shifting, emotional control and self-monitoring) and a metacognition index (initiation, working memory, planning/organizing, task monitoring and of materials). The answers to this self-administered questionnaire are given in a three-point Likert scale format (never/sometimes/often). A global executive composite (GEC) will be formed by the total score. The test has been found to be reliable (an internal consistency of α .80-.94 for the clinical scales; α .96-.98 for the indexes) [50].

##### Feasibility

- *Adherence:* Willingness to participate in the study (participation/refusal, yes, no); dropout for any reason (yes/no); involvement in the interventions for 8 weeks period (yes/no)
- *Usability:* “Was the game usable?” (yes/no)
- *Satisfaction:* “Have you been satisfied with the game?” (yes/no)
- *Use:* “Would you like to use the game in the future?” (yes/no/maybe)

Background information including socio-demographic characteristics and illness history will be collected (age, gender, marital status, level of education, employment status, living situation, illness history, illness history, current digital game playing (hours a week). If needed, detailed information about each participant’s diagnosis and the time of traumatic brain injury will be collected based on electronic medical records to limit the amount of information to be collected from patients themselves.

#### Time for the Data Collection and Follow-Up

Patient data will be collected at three different times: at baseline, after the intervention (8 weeks), and as a follow-up, 3 months after the intervention. If required by the participant, he or she will receive a text message about 2 days before the meeting to remind them of the coming follow-up meeting.

#### Sample Size

The calculations for the sample size needed in each group are preliminary estimations to guide our data collection, which are based on previous studies: (1) TMT (version A) and (2) depression (PHQ-9). First, if a score on the TMT version will be about 71, the mean change in the scores during the follow-up will be 30, and standard deviation of the TMT scores will be 53 [41]. This difference between groups could be expected to be significant (with a power of 85%, *p*=.05) if the sample size in each group is 30 subjects. Second, if the average level of the PHQ-9 score is about 10, the mean change in the scores during the follow-up is then 3 (SD 5) [48]. The difference could be expected to be significant (with a power of 85%, *p*=.05) if the sample size in each group is 27 subjects. Thus, based on these preliminary power calculations, the sample size to be used in this study (30 in each group) is not very strong but will be reasonable for a feasibility study aiming to detect preliminary changes within the group, between baseline and follow-up outcome measurements. However, this means that the attrition rate of the study should be near 0%. The outcomes of this study will be used as a guide to estimate a sample size for a multi-center study to be conducted, hopefully in the future.

#### Randomization

The study will be individually randomized. The randomization and patient allocation will be fully centralized. A central randomization service provided by the University of Turku will be used. Allocation will be computer generated. The participants will be randomly assigned (a block randomization in three blocks) using numbers via computerized assignment developed by the independent trial statistician and implemented by the Research Assistants (2), who are trained in patient randomization and data collection.

The research assistants overseeing patient recruitment and randomization will be aware of the assignments. Allocation will not be masked to participants in intervention and control groups or Research Assistants who will recruit patients. The psychologists, as cognitive outcome assessors, will be masked. The data analyst (the trial statistician) will be kept blinded to the allocation. As far as we are aware, there will be no contact between participants in different groups; participants will come from a wide geographic area in the catchment area of the Southwest Hospital District in Finland.

#### Analysis Plan

All the participants will be analyzed at the baseline, after 8 weeks when the intervention has been finalized, and at 3 months, using all the scales for primary and secondary outcomes. Comparisons in possible differences between means in 3 intervention groups and 3 time points will be conducted using analysis of variance (ANOVA test). Possible post hoc tests may be performed using paired *t*-tests, or Chi square tests to detect significant differences before and after treatment in sub-groups, if needed. Further, significant differences between groups will be evaluated applying the unpaired Student’s *t*-test using the conventional 95% level of confidence. Statistical analyses will be done using SAS System for Windows, version 9.4 (SAS Institute Inc.). *P* values less than 0.05 will be considered statistically significant.

The cumulative monitoring data during the 8-week period regarding gaming for the total number of received parameters (frequency, timing, time) will be calculated. Feasibility information (secondary outcomes) available for each patient will be calculated (participation/refusal rate, measurement instrument filled, drop-outs, acceptability, usability, satisfaction, willingness) and compared to the highest possible representative numbers (100% participation rate; measurement instrument filled, drop-out rate 0%, acceptability 100%, usability 100%, satisfaction 100%, willingness to use games in the future 100%).

Criteria for feasibility are as follows:

- Willingness to participate 80% (refusal rate 20%)
- We expect that more than 75% of the prescheduled measurements will be performed (adherence).
- Less than 25% of participants drop out for to any reason (adherence)
- The acceptability of the game use is at least 95% (in intervention and control groups) (adherence).
- Usability evaluation for the gaming system is at least 80% (1 item; dichotomous scale yes/no) (usability).
- More than 80% are satisfied with the games (1 item; dichotomous scale, yes/no) (satisfaction)
- We expect that 60% are willing to use the games later as part of their recovery process (use in the future).

#### Ethical Issues

A proposal for this study has been evaluated by the ethical committee of the Turku University Hospital (ETMK 41/1801/2015). The permission to conduct the study has been granted by the Turku University Hospital (T89/T04/008/2015). The trial has been officially registered (NCT02425527). All patients will be informed of the study orally and in written format. Informed consent will be obtained for experimentation with human subjects. The neurologist is a part of the study group and an expert in this kind of treatment. Study participation is completely voluntary (a signed informed consent, the Declaration of Helsinki 2013).

Possible ethical issues have already been taken into account in the development phase of the study: the rehabilitation and entertainment games were pre-tested with five healthy adults and with five people with TBI. Based on pre-tests, it was possible to identify more specific inclusion and exclusion criteria for the study. In addition, specific games were identified and excluded, (eg, those which may cause dizziness or headaches in participants due to dark colors, 3D tunnel effects, and so on) [51].

Study participants will have full understanding of the content and purposes of the study. They will be informed orally a total of 3 times before their informed consent is asked for (2 telephone calls, 1 face-to-face meeting). Written consent to participate in the study will be requested from participants after they have been informed of all aspects of the study. According to the Finnish Medical Research Act (488/1999) [52] the participants will be informed that in case they withdraw from the study for any reason, their information that has already been collected will not be removed from the research material. Participants will have no obligation to provide information they do not want to share. They can also withdraw from the study at any time without providing a reason. Refusal to participate in the study will not have any effects on their daily lives. Further, the psychological safety and emotional stability of the responders also will be taken into account. They will be free to stop answering questionnaires at any time, in case they feel uncomfortable or distressed. If a participant would like to have additional information, they will be informed about a contact person who can answer their questions by telephone, email or text messaging.

To shorten the first face-to-face meeting with the Research Assistant, participants will receive paper-format questionnaires by post. For follow-up interviews (after the 8-week intervention and 3-month period) participants will return the filled questionnaires in a pre-paid envelope or during their face-to-face meeting with the Research Assistant. The participants in the “as usual” group will return the filled-in questionnaires by post only. The participants will not be charged for any costs associated with data collection. Participants will be informed that if the console breaks down, they are not liable for damages. In special cases, the Technical Assistant will visit the participant’s home to repair devices if needed. If any technological problems arise during the intervention, the participants have the opportunity to contact the Technical Assistant by phone or email to receive practical or hands-on support.

The privacy rights of human subjects will be observed at all times. Respondents’ answers will not be provided to organizations outside the study. Information obtained will be used only for its original purpose. Information derived from research data will be coded and recorded in such a way that subjects cannot be identified [53]. All collected data will be transferred, recorded and securely stored (Personal Data Act 523/1999, Archive Act 831/1994, Constitutional Act 731/1999) [54-56].

The participants in the group assigned entertainment gaming may choose games targeted for an audience of young adolescents or 16 years old or younger. All participants in intervention groups can change the game during the intervention period if they find the game to be unpleasant, boring or otherwise dissatisfying. Moreover, all participants will be adults (over 18 years) who are capable of playing the games if they are willing to do so. The games being used are commercially available; however, some are designed only for adult players. These action games may include explicit graphic content, emotionally challenging themes or rough language. We found studies related to possible outcomes of playing this type of action game: some studies concluded that action games with violent elements may cause negative outcomes with aggression [57,58]. Contrasting results have also been found: it has been concluded, for example, that action games do not lead to aggressive behavior in adult users [59]. Regarding the potential positive effects of the games, they are associated with higher visuospatial cognition or visual skills [59]. Non-violent games do not appear to generalize to visuospatial cognitive abilities even when they involve visual rotation tasks [60].

The study will be conducted in accordance with the Finnish Medical Research Act (488/1999, amended 295/2004) [54], and Amendments (295/2004); Personal Data Act (523/1999) [54]; the Act on the Openness of Government Activities (621/1999). The principles of research ethics will be followed [61,62]. Any regulations addressing the conduct of a vulnerable population will be taken into account. This population could not be reached in another data collection method (eg, a register study).

## Abbreviations

ANOVA: analysis of variance
BRIEF-A: Behavior Rating Inventory of Executive Function – Adult Version
GSC: General Self-efficacy Scale
PASAT: Paced Auditory Serial Addition Test
PHQ-9: Patient Health Questionnaire
PS3: Playstation 3
TBI: traumatic brain injury
TEKES: The Finnish Funding Agency for Technology and Innovation
TMT: Trail Making Test
WAIS-IV: Wechsler Adult Intelligence Scale, 4th Edition
